# Recurrence or persistence of atrial fibrillation is associated with frailty and adverse outcomes: the SAGE-AF cohort study

**DOI:** 10.1186/s12877-026-07794-z

**Published:** 2026-06-13

**Authors:** Bahjat Z Ghazzal, Darleen Lessard, Joseph Tejan, Edith M Otabil, Adam Doerr, Nouran Y Nagy, Daniel J Harrington, Matheus Montenario, Jane S Saczynski, David D McManus

**Affiliations:** 1 Department of Medicine, University of Massachusetts Chan Medical School, 55 Lake Avenue North, Worcester, MA 01655 USA; 2 Department of Population and Quantitative Health Sciences, University of Massachusetts Chan Medical School, Worcester, MA USA; 3 Division of Cardiology, Department of Medicine, University of Massachusetts Chan Medical School, Worcester, MA USA; 4 University of New England College of Osteopathic Medicine, Biddeford, ME USA; 5 Division of Cardiology, Department of Medicine, Saint Vincent Hospital, Worcester, MA USA; 6 Department of Medicine, Saint Rita’s Medical Center, Lima, OH USA; 7 Department of Pharmacy and Health System Sciences, Northeastern University, Boston, MA USA

**Keywords:** Atrial Fibrillation, Frailty, Recurrence, Persistence, Major Bleeding

## Abstract

**Background:**

Atrial fibrillation (AF) is a prevalent arrhythmia in older adults. While traditional risk factors are well-established, the role of frailty, a marker of biological aging, in the course of AF over time remains underexplored.

**Methods:**

We utilized data from 854 older adults (≥ 65 years) with AF and a CHA_2_DS_2_-VASc score ≥ 2, enrolled in the Systematic Assessment of Geriatric Elements (SAGE-AF) prospective cohort study, who had electrocardiograms (ECGs) available at both a baseline and two-year follow-up visit. Participants were classified as having AF recurrence if baseline ECG showed sinus rhythm and year-two ECG showed AF, and as having AF persistence if AF was present at both time points.

**Results:**

We included 773 older adults in our analysis (mean age 76 ± 7, 52% male, 92% white). AF recurrence or persistence was detected on ECG in 379 (49%) of participants at two-year follow-up. Baseline frailty or pre-frailty was significantly associated with higher odds of AF recurrence or persistence (aOR 1.52, 95%CI 1.03–2.25). Participants with recurrent or persistent AF had a higher incidence of major bleeding events, even after adjustment for anticoagulation, age, and other cardiovascular risk factors (aHR 1.87, 95%CI 1.01–3.45).

**Conclusion:**

Frailty or pre-frailty increases odds of AF recurrence or persistence at two years, underscoring the potential role of biological aging in influencing the course of AF over time. AF recurrence or persistence was associated with greater hazards of major bleeding, even after adjustment for key clinical factors, suggesting residual risk not captured by measured covariates.

## Key Points

• Among older adults with atrial fibrillation, baseline frailty or pre-frailty was associated with recurrence or persistence of atrial fibrillation on electrocardiogram at two-year follow-up.

• Recurrence or persistence of atrial fibrillation was associated with an increased risk of major bleeding, even after adjustment for anticoagulation and traditional risk factors.

## Impact statement

We certify that this work presents novel clinical research. This study identifies a novel association between baseline frailty or pre-frailty and recurrence or persistence of atrial fibrillation on follow-up electrocardiogram, beyond traditional cardiovascular risk factors. These findings highlight the importance of biological aging in atrial fibrillation over time and support routine frailty assessment in older adults with this condition, including the development of scalable assessment methods that can be deployed across clinical settings.

## Introduction

Atrial fibrillation (AF) is the most common sustained cardiac arrhythmia in the United States [1]. AF is disproportionately a disease of older persons and it affects nearly one in thirteen individuals of those aged 65 years and older [1, 2]. The natural progression of AF typically involves advancement from paroxysmal to persistent or permanent forms, driven by pathological electrical and structural remodeling [3]. Progression to more sustained forms of AF has been linked to increased adverse cardiovascular (CV) outcomes and all-cause mortality [4–7]. Recently updated AF treatment guidelines emphasize addressing CV risk factors that contribute to subclinical atrial remodeling for secondary prevention [8].

In addition to age, the duration and intensity of exposure to CV risk factors over adulthood are associated with greater lifetime risk for incident and recurrent AF [9]. As a result, a global hypothesis has emerged that underlying CV risk factor burden drives pathological remodeling and increases the risk of AF complications [10]. This hypothesis has been supported by studies showing that echocardiographic markers of atrial enlargement and measures of atrial and ventricular stress, including atrial and b-type natriuretic peptide, are strongly tied to incident and recurrent AF [11, 12]. An emerging body of literature now suggests that frailty captures components of biological age not reflected in traditional risk factor assessments, inclusive of lifetime weathering effects such as stress exposure, poor sleep, and physical inactivity [13, 14]. More recently, frailty has been conceptualized within a broader construct of “clinical complexity” that also encompasses multimorbidity and polypharmacy, each of which contributes additively to adverse outcomes in patients with AF [15].

Utilizing data from the Systematic Assessment of Geriatric Elements in Atrial Fibrillation (SAGE-AF) prospective cohort study [16], we sought to examine the relationship of baseline frailty or pre-frailty and other traditional risk factors with recurrence or persistence of AF at the end of a two-year follow-up period among a population of older patients with history of AF. We also investigated whether patients with recurrent or persistent AF at year-two follow-up were more likely than those who maintained sinus rhythm (SR) from baseline to follow-up, or who experienced restoration of SR from AF at baseline, to experience adverse outcomes including hospitalization, bleeding events, and stroke. This investigation focuses on the hypothesis that frailty or pre-frailty captures residual risk for AF course over time beyond traditional CV risk factors.

## Methods

### Study sample

The SAGE-AF study is a prospective cohort study of 1244 patients with diagnosed non-valvular AF enrolled from one of five medical centers in Massachusetts and Georgia between June 2016 and August 2018; the design, recruitment, and baseline characteristics have been previously described in detail [16]. Inclusion criteria were age *≥* 65 years, scheduled for an outpatient visit, diagnosed with non-valvular AF (NVAF) confirmed by ECG, Holter monitoring, or medical records, and a CHA_2_DS_2_-VASc score of *≥* 2. Exclusion criteria included documented absolute contraindication to oral anticoagulant (OAC) therapy, use of OAC for conditions other than NVAF, scheduled for a high-risk bleeding invasive procedure, lack of capacity to provide informed consent, non-English speaking, unwillingness to return for follow-up visits, pregnancy, or incarceration. All participants provided written informed consent prior to their scheduled clinic visit. Study protocols were approved by the Institutional Review Board at the University of Massachusetts Chan Medical School (#H-00009079) in addition to those at other participating enrollment sites.

### Medical record abstraction

Trained study staff extracted demographic, clinical, treatment, and laboratory data from participants’ medical records. Baseline comorbidities including hypertension (HTN), heart failure (HF), myocardial infarction (MI), diabetes (DM), and chronic kidney disease (CKD) were recorded, along with medication prescriptions including oral anticoagulant use, anticoagulant subtype (warfarin vs. direct-acting oral anticoagulant [DOAC]), and concomitant antiplatelet therapy at enrollment. The type of AF treatment strategy was categorized as rhythm or rate control by study staff using a consistent rubric. Rhythm control was defined as having at least one of the following: ablation or cardioversion prior to enrollment, or use of an antiarrhythmic drug at the baseline study visit. All other participants were considered to be treated with rate control. AF category at baseline was abstracted from clinical notes and categorized as paroxysmal, persistent or permanent AF.

### ECG analysis and interpretation

**Fig. 1:**
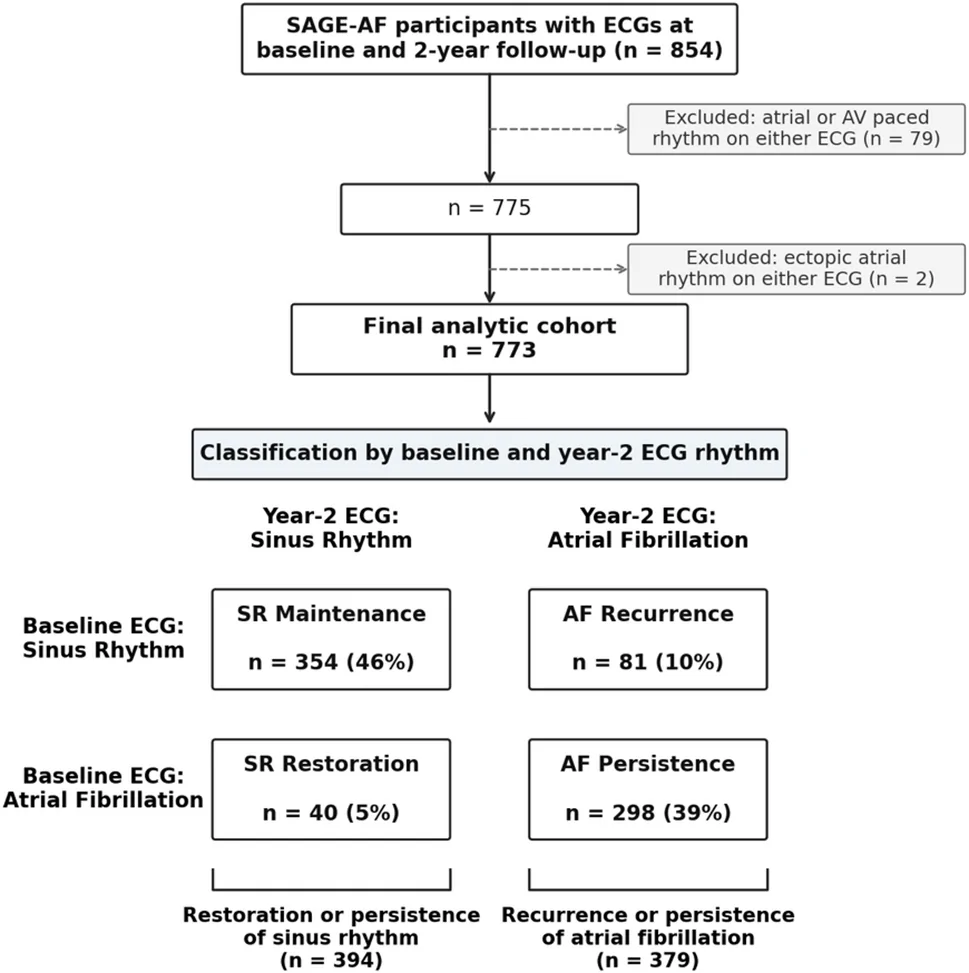
Participant flow diagram and classification by baseline and year-two ECG findings. Of 854 SAGE-AF participants with ECGs available at both the baseline and two-year follow-up visits, 79 were excluded for atrial or atrioventricular paced rhythm and 2 for ectopic atrial rhythm, yielding a final analytic cohort of 773. Participants were classified into one of four categories by ECG rhythm at baseline and at two-year follow-up: SR maintenance, AF recurrence, SR restoration, and AF persistence, and grouped for analysis as restoration or persistence of sinus rhythm (*n* = 394) versus recurrence or persistence of atrial fibrillation (*n* = 379). Abbreviations: AF, atrial fibrillation; AV, atrioventricular; ECG, electrocardiogram; SAGE-AF, Systematic Assessment of Geriatric Elements in Atrial Fibrillation; SR, sinus rhythm

For this analysis, we used data from a sub-cohort of 854 SAGE-AF participants who were enrolled at UMass Memorial Medical Center and for whom ECGs were available at both the baseline and two-year follow-up visits. ECGs were abstracted and the rhythm coded by a board-certified cardiologist; the result was subsequently verified by a physician on the study team. In cases where ventricular pacing was present, the underlying atrial rhythm was determined and the ECGs were coded accordingly. ECGs showing atrial pacing or atrial arrhythmias other than AF were excluded from the analysis, as device-based algorithms that minimize of modify atrial and ventricular pacing have been shown to alter the natural history of AF and would have constituted a distinct exposure incompatible with our ECG-defined classification [17]. Individuals found to have atrial flutter on ECG were categorized as AF. Participants were classified into four categories based on baseline and follow-up ECG findings (Fig. 1): AF recurrence (baseline SR, year-two AF), AF persistence (AF at both visits), SR maintenance (SR at both visits), and SR restoration (baseline AF, year-two SR).

### Geriatric examination

SAGE-AF participants completed a comprehensive interview including a six-component geriatric assessment with validated measures for frailty, cognitive function, social support, depressive symptoms, vision, and hearing. Frailty was assessed using the Cardiovascular Health Survey (CHS) frailty scale, evaluating weight loss, exhaustion, low physical activity, slow gait speed, and weakness, with a score ranging from 0 to 5 [18]. Participants were classified as frail (three or more criteria), pre-frail (one or two criteria), or not frail (none). Cognitive function was assessed with the Montreal Cognitive Assessment Battery (MoCA), using a cut-off score of 23 for cognitive impairment[ 19, 20]. Depressive symptoms were measured with the nine-item Patient Health Questionnaire (PHQ-9), with a score of *≥* 5 indicating depressive symptoms [21]. Anxiety was assessed using the Generalized Anxiety Disorder (GAD-7) [22].Quality of life related to AF was evaluated with the twenty-item Atrial Fibrillation Effect on Quality-of-life (AFEQT) questionnaire, with scores < 80 indicating compromised quality of life [23]. Comorbidity burden was measured using the Charlson Comorbidity Index, which aggregates scores for various conditions to predict ten-year mortality risk; this index was calculated and updated by trained staff from medical record data to reflect changes in health status [24].

### Outcomes over two years of follow-up

Participants were followed for two years post-enrollment, with study examinations at one- and two-year intervals. Trained study staff abstracted outcome data, including hospitalizations, strokes, and bleeding events, from participants’ medical records. Outcomes were adjudicated by a committee of three expert clinicians. Major bleeding events were defined as symptomatic bleeding in a critical area or organ, a hemoglobin drop of > 2 g/dL, transfusion of *≥* 2 units of whole blood, or resulting in permanent disability. Clinically relevant non-major (CRNM) bleeding events were defined as overt bleeding requiring medical intervention, unscheduled physician contact, temporary interruption of anticoagulation, pain, or impairment of daily activities. Strokes were classified based on imaging studies (CT or MRI) and clinical notes describing neurological deficits, with ischemic stroke defined as a new sudden focal neurological deficit from a presumed cerebrovascular cause that is not reversible within 24 h, and transient ischemic attacks defined as events lasting less than 24 h. Hospitalizations were classified as cardiovascular, bleeding, or other, and only unplanned hospitalizations were considered outcomes. Participants were considered to have such an outcome if any of these events occurred at least once during the follow-up period.

### Statistical analysis

Participant characteristics at baseline were compared between those with persistent or recurrent AF and those with maintained or restored SR at the two-year study visit using analysis of variance for continuous variables and chi-square tests for categorical variables. Most covariates had minimal missing data (< 2%); the exception was baseline AF type, which was missing for 96 participants (12.4%) and was the prinicipal contributor to the reduced sample available for fully adjusted models (*n* = 665). Analyses used complete cases. We calculated the odds of having persistent or recurrent AF at two-year follow-up for all baseline clinical and demographic variables and geriatric components. Univariate analysis was performed first to identify significant predictors. Variables found to be significant in univariate analysis were subsequently included in a multivariate analysis using a Generalized Estimating Equations (GEE) model to yield adjusted odds ratios (ORs). Multivariate models included factors associated with persistence or recurrence of AF on year-two follow-up ECG in univariate models, including age, female sex, college-graduate status, AF duration, baseline AF type (paroxysmal, persistent, or permanent), rate versus rhythm control, anticoagulation (warfarin, DOAC, or none), heart failure, hypertension, peripheral vascular disease, renal disease, CHA₂DS₂-VASc score, Charlson Comorbidity Index, current smoking, cognitive impairment, frailty or pre-frailty, and AFEQT score < 80. To address whether associations differed by sex, models were additionally fit separately in men and women, and formal sex × frailty interaction terms were tested for both the year-two AF outcome and the major-bleeding outcome. The incidence of adverse clinical outcomes during the two-year follow-up period was compared between groups using analysis of variance. We additionally ran competing-risk proportional hazards models (with death as a competing event) for each adverse outcome in Table 3 at year-two AF status as the key predictor in both unadjusted models and adjusted models, controlling for the significant factors from Table 2. In a sensitivity analysis, these outcome models were re-fit with anticoagulation modeled as warfarin, DOAC, or none (Supplementary Table S3).

## Results

Of the 854 participants included in the primary analysis, 79 were excluded due to atrial or atrioventricular paced rhythms on either ECG, and 2 were excluded due to ectopic atrial rhythm on either ECG, resulting in a final cohort of 773 participants (Fig. 1). The average age of participants was 75.5 (SD 7.3) years; 52% (*n* = 400) were male and 92% (*n* = 709) were white. Demographic and clinical characteristics by group are presented in Table 1.

**Table 1:** Baseline characteristics of older adults with atrial fibrillation who experienced restoration or persistence of sinus rhythm compared with those who experienced recurrence or persistence of atrial fibrillation on ECG at the two-year follow-up visit

	Participants with restoration or persistence of sinus rhythm at two-year follow-up (*n* = 394)	Participants with recurrence or persistence of atrial fibrillation at two-year follow-up (*n* = 379)	***p*** **-value**
Demographic			
Age (mean, yrs. (sd))	73.8 (6.6)	77.3 (7.4)	**< 0.001**
Sex (%)			0.09
Male	48.7	54.9	
Female	51.3	45.1	
Race (%)			0.44
White	91.1	92.6	
Non-White	8.9	7.4	
Married (%)	60.2	51.7	0.20
College Graduate or Higher (%)			**0.01**
* ≤* College	44.3	54.3	
College Graduate	55.7	45.7	
**Clinical**			
AF Type (%)			**< 0.001**
Paroxysmal	87.9	35.7	
Persistent	10.4	50.2	
Permanent	1.7	14.2	
Treatment (%)			**< 0.001**
Rhythm Control (ablation or cardioversion or anti-arrhythmic drug therapy)	57.6	43.3	
Rate Control (all others)	42.4	56.7	
On Warfarin or Other Anticoagulant (%)	76.1	91.6	**< 0.001**
AF Duration (mean, yrs (sd))	3 (7)	6 (8)	**< 0.001**
CHA_2_DS_2_-VASc (mean (sd))	4.01 (2.5)	4.5 (2.7)	**< 0.001**
HASBLED (mean (sd))	3.2 (3.0)	3.3 (5.0)	**0.047**
Charlson Index (mean (sd))	4.5 (4.0)	6.2 (4.1)	**< 0.001**
Heart Failure (%)	22.1	43.8	**< 0.001**
Myocardial Infarction (%)	17.3	18.2	0.73
Hypertension (%)	86.0	92.6	**< 0.001**
Peripheral Vascular Disease (%)	11.2	18.7	**0.003**
Renal Disease (%)	21.3	32.7	**< 0.001**
Type 2 Diabetes Mellitus (%)	23.6	24.5	0.76
Anemia (%)	29.7	32.5	0.41
Chronic Lung Disease (%)	22.3	24.5	0.47
Major Bleeding (%)	18.0	20.6	0.37
Stroke (%)	8.4	11.4	0.17
**Psychosocial & Geriatric**			
Cognitive Impairment^a^ (%)	28.7	43.5	**< 0.001**
AFEQT Score < 80 (%)	37.8	41.4	0.31
Baseline Frailty or Pre-Frailty^b^			**< 0.001**
Frail (*≥* 3 criteria met)	8.9	12.4	
Pre-Frail (1 or 2 criteria met)	44.7	58.3	
Not Frail (no criteria met)	46.5	29.3	
Baseline Anxiety^c^ (%)	22.1	19.5	0.38
Baseline Depression^d^ (%)	24.0	27.0	0.42
**Health Behaviors**			
Alcohol Use (%)	34.0	28.5	0.10
Smoking Status (%)			**0.032**
Never Smoker	48.5	45.7	
Former Smoker	47.5	53.0	
Current Smoker	4.1	1.3	

Values are the percentage of participants experiencing each outcome at least once during follow-up; p-values are from chi-square tests. Bold indicates statistical significance at α = 0.05

*Abbreviations*: *AF *Atrial fibrillation, *CRNM *Clinically relevant non-major, *ECG *Electrocardiogram, *SR *Sinus rhythm

At the two-year follow-up, AF was detected on ECG in 49% (*n* = 379) of participants. Among them, 10% (*n* = 81) had SR at baseline and demonstrated recurrence of AF at the two-year follow-up, while 39% (*n* = 298) had AF at baseline and showed persistence of AF at follow-up. Additionally, 5% (*n* = 40) of participants experienced restoration of SR, while 46% (*n* = 354) had maintenance of SR. Baseline characteristics differed significantly between those with persistent or recurrent AF and those with maintained or restored SR on ECG at the two-year visit. The AF group was older (77.3 vs. 73.7 years, *p* < 0.0001), had carried a diagnosis of AF for a longer period (6 vs. 3 years, *p* < 0.0001), and was less likely to be on rhythm control (43.3% vs. 57.6%, *p* < 0.0001). As shown in Table 1, participants with recurrent or persistent AF had higher mean CHA_2_DS_2_-VASc (4.5 vs. 4.0, *p* < 0.0001) and HAS-BLED (3.3 vs. 3.2, *p* = 0.0468) scores, indicating greater stroke and bleeding risk. As expected, a greater proportion of individuals had persistent or permanent AF in the AF group (35.7% paroxysmal, 50.2% persistent, 14.2% permanent) compared with the SR group (87.9% paroxysmal, 10.4% persistent, 1.7% permanent). With respect to geriatric conditions, frailty or pre-frailty was significantly more prevalent in the AF group (70.71% vs. 53.55%, *p* < 0.0001), as was cognitive impairment (43.5% vs. 28.7%, *p* < 0.0001).

**Fig. 2:**
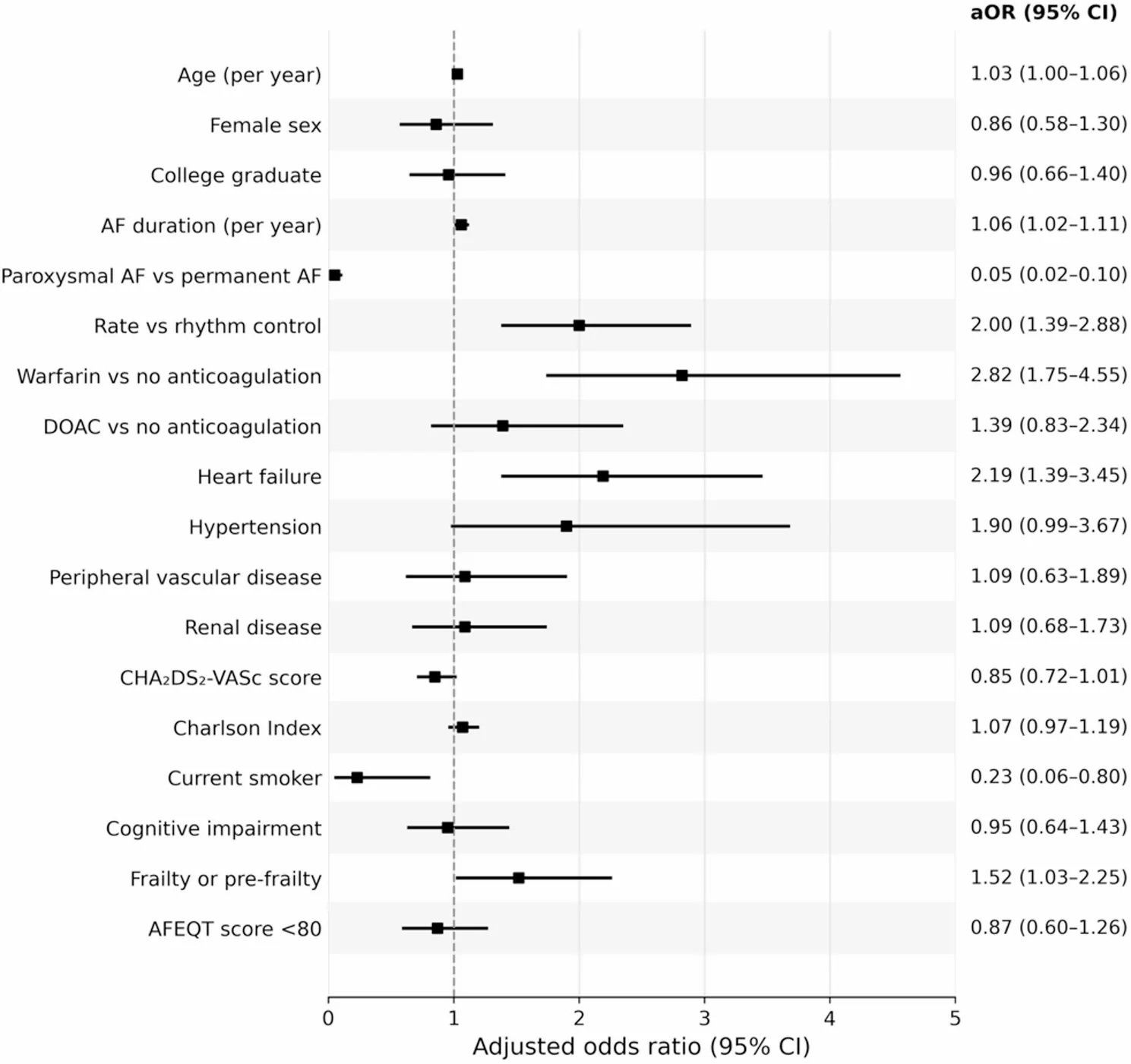
Adjusted odds of recurrence or persistence of atrial fibrillation at two years. Forest plot of the adjusted odds ratios for each predictor in the multivariable generalized estimating equations model (corresponding to Table 2). Squares denote the point estimate and horizontal lines the 95% confidence interval; the adjusted odds ratio and 95% confidence interval for each predictor are listed at right. The vertical dashed line marks the null (odds ratio = 1). Estimates whose 95% confidence interval excludes 1 are statistically significant at α = 0.05. Adjusted for age, female sex, college-graduate status, AF duration, baseline AF type (paroxysmal, persistent, or permanent), rate versus rhythm control, anticoagulation (warfarin, DOAC, or none), heart failure, hypertension, peripheral vascular disease, renal disease, CHA₂DS₂-VASc score, Charlson Comorbidity Index, current smoking, cognitive impairment, frailty or pre-frailty, and AFEQT score < 80. Abbreviations: aOR, adjusted odds ratio; AF, atrial fibrillation; AFEQT, Atrial Fibrillation Effect on Quality-of-Life questionnaire; CHA₂DS₂-VASc, congestive heart failure, hypertension, age ≥75 years (doubled), diabetes mellitus, prior stroke/transient ischemic attack/thromboembolism (doubled), vascular disease, age 65–74 years, sex category (female); CI, confidence interval; DOAC, direct-acting oral anticoagulant

In models adjusted for factors associated with persistent or recurrent AF in univariate analyses, AF duration at baseline (aOR 1.06, 95%CI 1.02–1.11) and the presence of heart failure (aOR 2.19, 95%CI 1.39–3.45) were significantly associated with higher odds of persistence or recurrence of AF at two years. With anticoagulation modeled by subtype, warfarin use was strongly associated with recurrent or persistent AF (aOR 2.82, 95%CI 1.75–4.55), whereas DOAC use was not significantly different from no anticoagulation (aOR 1.39, 95%CI 0.83–2.34). Associations between age (aOR 1.03, 95%CI 1.00–1.06) and hypertension (aOR 1.90, 95%CI 0.99–3.67) with persistent or recurrent AF versus maintained or restored SR at two-years were attenuated after multivariate adjustment (Table 2; Fig. 2A). Regarding geriatric-specific factors, frailty or pre-frailty at baseline significantly increased odds of persistence or recurrence of AF in both univariate (OR 2.03, 95%CI 1.54–2.67) and multivariate analyses (aOR 1.52, 95%CI 1.03–2.25). In sex-stratified models the adjusted association was significant in men (aOR 1.98, 95%CI 1.16–3.39) and attenuated in women (aOR 1.34, 95%CI 0.72–2.49); however, a formal sex × frailty interaction was not significant (*p* = 0.33), indicating that the data do not support a true difference in the frailty–AF association between sexes. Baseline cognitive impairment was associated with increased odds of persistent or recurrent AF in univariate models (OR 1.75, 95%CI 1.33–2.29), but this association was attenuated in multivariate analysis (aOR 0.95, 95%CI 0.64–1.43).

**Table 2:** Adjusted and unadjusted odds of recurrence or persistence of atrial fibrillation versus restoration or persistence of sinus rhythm on ECG at two years among older adults with atrial fibrillation

	**Unadjusted Odds of AF Recurrence or Persistence (95% CI)**	Adjusted Odds of AF Recurrence or Persistence* (95% CI)
Age	**1.07 (1.05, 1.09)**	
Female	**0.72 (0.56**,** 0.94)**	0.85 (0.58, 1.30)
Non-Hispanic White	1.49 (0.92, 2.41)	
College Graduate	**0.69 (0.53**,** 0.90)**	0.96 (0.66, 1.40)
AF Duration	**1.10 (1.06**,** 1.15)**	**1.06 (1.02**,** 1.11)**
Paroxysmal AF vs. Permanent AF at baseline exam	**0.02 (0.01**,** 0.06)**	**0.05 (0.02**,** 0.10)**
Persistent AF vs. Permanent AF at baseline exam	0.41 (0.17, 1.01)	
Rate Control vs. Rhythm Control	**1.85 (1.42**,** 2.41)**	**2.00 (1.39**,** 2.88)**
Warfarin vs. No Anticoagulation	**5.32 (3.50**,** 8.08)**	**2.82 (1.75**,** 4.55)**
DOAC vs. No Anticoagulation	**2.11 (1.33**,** 3.33)**	1.39 (0.83, 2.34)
Alcohol Use	0.79 (0.59, 1.04)	
Anemia	1.12 (0.85, 1.48)	
Chronic Lung Disease	1.06 (0.78, 1.44)	
Type 2 Diabetes	1.08 (0.80, 1.47)	
Heart Failure	**2.80 (2.11**,** 3.71)**	**2.19 (1.39**,** 3.45)**
Hypertension	**1.93 (1.21**,** 3.06)**	1.90 (0.99, 3.67)
Major Bleed	1.07 (0.77, 1.48)	
MI	1.11 (0.79, 1.56)	
PVD	**1.68 (1.18**,** 2.41)**	1.09 (0.63, 1.89)
Renal Disease	**1.72 (1.29**,** 2.31)**	1.09 (0.68, 1.73)
Stroke	1.21 (0.78, 1.88)	
CHA_2_DS_2_-VASc	**1.21 (1.11**,** 1.31)**	0.85 (0.72, 1.01)
HAS-BLED	1.09 (0.97, 1.23)	
Charlson Index	**1.16 (1.10**,** 1.23)**	1.07 (0.97, 1.19)
Current Smoker	**0.35 (0.14**,** 0.83)**	**0.23 (0.06**,** 0.80)**
Cognitive Impairment^a^	**1.75 (1.33**,** 2.29)**	0.95 (0.64, 1.43)
Frailty or Pre-frailty^b^	**2.03 (1.54**,** 2.67)**	**1.52 (1.03**,** 2.25)**
AFEQT < 80	**1.31 (1.01**,** 1.71)**	0.87 (0.60, 1.26)
Anxiety^c^	0.90 (0.66, 1.25)	
Depression^d^	1.18 (0.88, 1.59)	

Odds ratios are from a generalized estimating equations model. Bold indicates statistical significance at α = 0.05. The adjusted model was fit on 665 participants with complete covariate data (baseline AF type missing in 12%)

*Abbreviations*: *AF *Atrial fibrillation, *AFEQT *Atrial Fibrillation Effect on Quality-of-Life questionnaire, *CHA₂DS₂-VASc *Congestive heart failure, hypertension, age ≥ 75 years (doubled), diabetes mellitus, prior stroke/transient ischemic attack/thromboembolism (doubled), vascular disease, age 65–74 years, sex category (female), *CHS *Cardiovascular Health Study, *CI *Confidence interval, *DOAC *Direct-acting oral anticoagulant, *HAS-BLED *Hypertension, Abnormal renal/liver function, stroke, bleeding history or predisposition, labile INR, elderly, drugs/alcohol, *MoCA *Montreal Cognitive Assessment, *OAC *Oral anticoagulant, *OR *Odds ratio

* Adjusted for age, female sex, college-graduate status, AF duration, baseline AF type (paroxysmal, persistent, or permanent), rate versus rhythm control, anticoagulation (warfarin, DOAC, or none), heart failure, hypertension, peripheral vascular disease, renal disease, CHA₂DS₂-VASc score, Charlson Comorbidity Index, current smoking, cognitive impairment, frailty or pre-frailty, and AFEQT score < 80

ᵃ Cognitive impairment defined as MoCA ≤ 23

ᵇ Frailty assessed with the CHS frailty scale: frail (≥ 3 criteria), pre-frail (1–2 criteria), or not frail (0 criteria)

ᶜ Anxiety defined as GAD-7 ≥ 5

ᵈ Depression defined as PHQ-9 ≥ 5

**Fig. 3:**
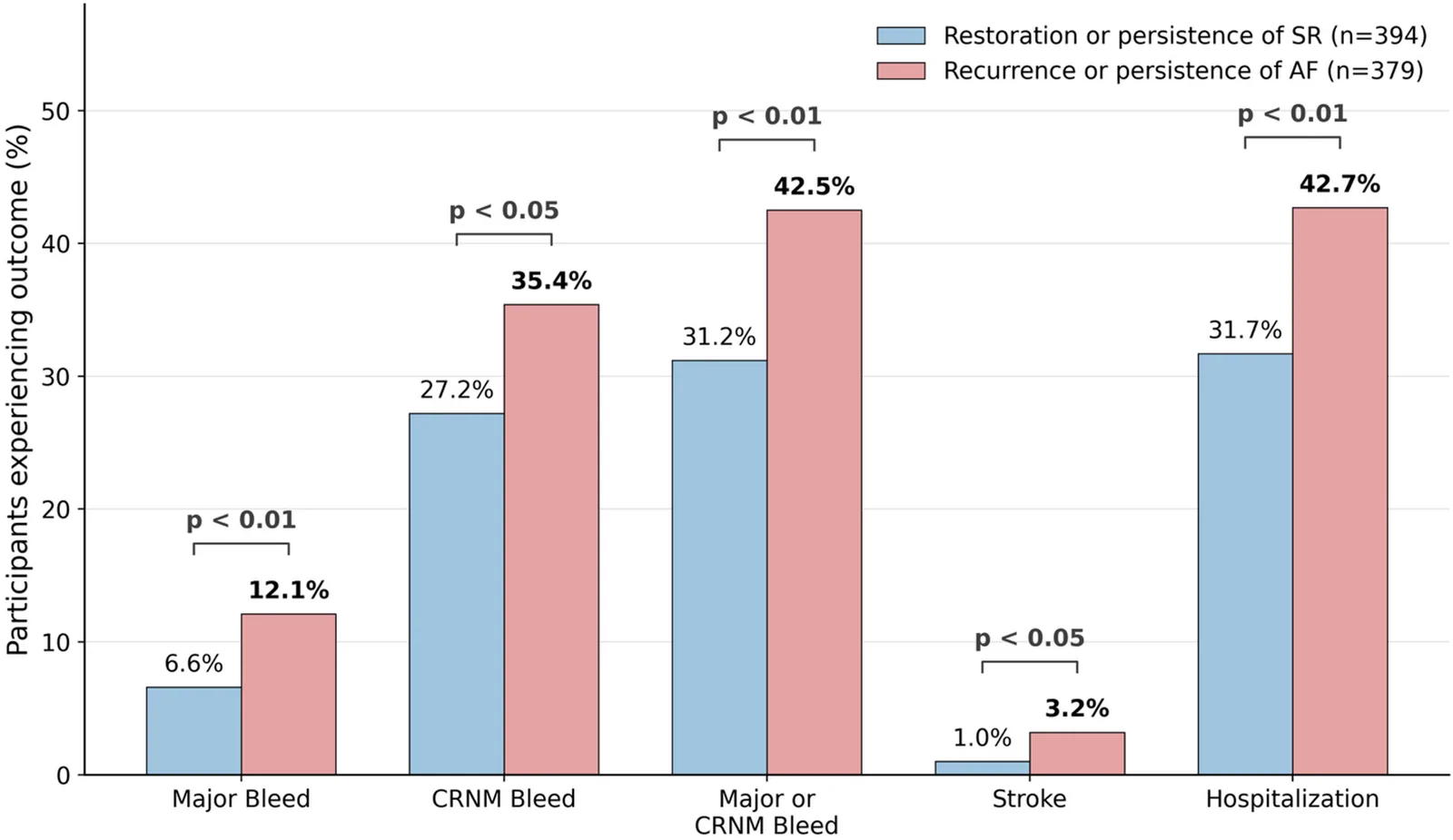
Adverse clinical outcomes over a two-year follow-up period, comparing participants with restoration or persistence of sinus rhythm (blue, *n* = 394) with those with recurrence or persistence of atrial fibrillation (red, *n* = 379) on ECG at the two-year follow-up visit. Bars represent the percentage of participants experiencing each outcome at least once during follow-up; brackets denote p-values from chi-square tests comparing the two groups. Abbreviations: AF, atrial fibrillation; CRNM, clinically relevant non-major; ECG, electrocardiogram; SR, sinus rhythm

Participants with recurrence or persistence of AF at the two-year follow-up were significantly more likely to experience any measured adverse clinical event during the follow-up period (Table 3; Fig. 3). The combined occurrence of major and clinically relevant non-major (CRNM) bleeding events was more common in the recurrent or persistent AF group than in the maintained or restored SR group (42.5% vs. 31.2%, *p* = 0.0012), as was major bleeding alone (12.1% vs. 6.6%, *p* = 0.0081) and CRNM bleeding alone (35.4% vs. 27.2%, *p* = 0.0139). Stroke was also more frequent in the AF group (3.2% vs. 1.0%, *p* = 0.0357), as was hospitalization (42.7% vs. 31.7%, *p* = 0.0015). Proportional hazards models showed a significant association between persistence or recurrence of AF on year-two ECG and adverse clinical outcomes, with the association for major bleeding remaining significant after adjustment for confounding variables (Table 4). This major-bleeding association was attenuated to borderline significance when anticoagulation was modeled by subtype (aHR 1.77, 95%CI 0.96–3.25); Supplementary Table S3), consistent with partial mediation through anticoagulation type. The sex × frailty interaction for major bleeding was also non-significant (*p* = 0.30); sex-stratified outcome estimates are provided in the Supplementary Tables S1 and S2.

**Table 3:** Adverse clinical outcomes over a two-year follow-up period among older adults with atrial fibrillation who experienced restoration or persistence of sinus rhythm compared with those who experienced recurrence or persistence of atrial fibrillation on ECG at the two-year follow-up visit

Clinical Outcomes	Participants with restoration or persistence of sinus rhythm at two-year follow-up (*n* = 394)	Participants with recurrence or persistence of atrial fibrillation at two-year follow-up (*n* = 379)	*p*-value
Major Bleed (%)	6.6	12.1	**< 0.01**
CRNM Bleed (%)	27.2	35.4	**< 0.05**
Major or CRNM Bleed (%)	31.2	42.5	**< 0.01**
Stroke (%)	1.0	3.2	**< 0.05**
Hospitalization (%)	31.7	42.7	**< 0.01**

Values are the percentage of participants experiencing each outcome at least once during follow-up; p-values are from chi-square tests. Bold indicates statistical significance at α = 0.05

*Abbreviations*: *AF *Atrial fibrillation, *CRNM *Clinically relevant non-major, *ECG *Electrocardiogram, *SR *Sinus rhythm

**Table 4:** Unadjusted and adjusted hazards of bleeding, stroke, or hospitalization over two years among older adults with atrial fibrillation who experienced recurrence or persistence of AF versus restoration or persistence of sinus rhythm on ECG at the two-year follow-up visit

Clinical Outcomes	Unadjusted HR (95% CI)	Adjusted HR* (95% CI)
Major Bleed	1.82 (1.12–2.94)	**1.87 (1.01–3.45)**
CRNM Bleed	**1.30 (1.01–1.68)**	1.07 (0.76–1.51)
Major or CRNM Bleed	**1.45 (1.15–1.84)**	1.23 (0.90–1.67)
Stroke	**3.15 (1.01–9.77)**	2.92 (0.63–13.47)
Hospitalization	**1.38 (1.09–1.74)**	1.07 (0.77–1.48)

Hazard ratios are from competing-risk proportional-hazards models with death as the competing event. Bold indicates statistical significance at α = 0.05

*Abbreviations*: *AF *Atrial fibrillation, *CI *Confidence interval, *CRNM *Clinically relevant non-major, *ECG *Electrocardiogram, *HR *Hazard ratio, *SR *Sinus rhythm

* Adjusted for the same covariate set as Table 2: age, female sex, college-graduate status, AF duration, baseline AF type (paroxysmal, persistent, or permanent), rate versus rhythm control, anticoagulation (warfarin, DOAC, or none), heart failure, hypertension, peripheral vascular disease, renal disease, CHA₂DS₂-VASc score, Charlson Comorbidity Index, current smoking, cognitive impairment, frailty or pre-frailty, and AFEQT score < 80. A sensitivity analysis modeling anticoagulant subtype is provided in Supplementary Table S3

## Discussion

Using data from a unique prospective cohort of older adults with well-characterized AF and rigorous follow-up examinations, baseline frailty or pre-frailty were strongly associated with greater odds of recurrence or persistence of AF at two years, even after adjustment for treatment, AF category, and CV risk factor burden. Furthermore, recurrence or persistence of AF at two years was associated with greater hazards of major bleeding over the follow-up period, and this association persisted after adjustment for important covariates including anticoagulation use and HAS-BLED score. Our findings suggest that frailty or pre-frailty captures elements of risk for the course of AF over time that are not fully reflected in traditional CV risk assessments.

Research from the Atherosclerosis Risk in Communities (ARIC) study [25], Cardiovascular Health Study (CHS) [26], and Framingham Heart Study (FHS)^27^ has reported a higher prevalence of AF among individuals classified as frail. Age has long been considered a primary driver of incident AF in older populations as a consequence of age-related left atrial fibrosis [28, 29]. Yet structural changes in atrial tissue have been linked to the presence and severity of AF, but not to chronological age [30], suggesting that age alone may not fully account for cardiac structural remodeling. Biological age—reflecting cellular aging influenced by lifestyle factors such as stress, sleep, and exercise—may therefore be central to risk of AF recurrence or persistence and related complications. Frailty, which assesses unintentional weight loss, self-reported exhaustion, weakness, slow walking speed, and low physical activity, correlates with the phenomenon of ‘weathering’ and may serve as a more accurate marker of accelerated aging and AF risk [14, 31]. Experimental models show that electrocardiographic markers of atrial remodeling are more strongly correlated with frailty than with chronological age [32], and findings from the Multi-Ethnic Study of Atherosclerosis link frailty to increased CV fibrosis and other markers of adverse cardiac remodeling [33].

Beyond frailty, our study identified age, duration of AF, hypertension, and heart failure, factors known to promote adverse cardiac remodeling, as associated with recurrence or persistence of AF at two years [34, 35]. Comorbidity burden, greater AF-related symptomatology, and lower quality of life were also associated with recurrent or persistent AF in univariate analyses. Although these did not reach statistical significance in multivariable models, they support the view that AF is a marker of systemic disease burden and that its course may be predicted by functional status and the number of comorbid non-cardiac conditions. This perspective is increasingly framed through the construct of “clinical complexity,” in which frailty, multimorbidity, and polypharmacy together define a phenotype that additively predicts adverse outcomes in AF cohorts [15]. Our findings support the position the biological-age markers such as frailty add prognostic information about AF course beyond conventional risk factors.

When anticoagulation was disaggregated into warfarin, DOAC, and none, the association with recurrent or persistent AF was driven almost entirely by warfarin, while DOAC use did not differ significantly from no anticoagulation. We interpret this as a reflection of treatment selection rather than a pharmacologic effect: by the enrollment period, DOACs had become first-line for most newly treated AF, so persistence on warfarin tended to mark patients with longstanding, more established AF—the same phenotype most likely to display AF on a follow-up ECG. Consistent with this, modeling anticoagulant subtype attenuated the association between recurrent or persistent AF and major bleeding from significant to borderline (Supplementary Table S3). Because warfarin carries a higher bleeding risk than DOACs [36], part of the apparent AF–bleeding association is likely mediated through anticoagulant type rather than reflecting a direct effect of rhythm status. Prior SAGE-AF analyses have characterized OAC prescribing patterns in this cohort in detail [16].

Importantly, frailty itself carries a bidirectional association with both ischemic stroke and major bleeding among patients with AF, and is among the most common reasons for which OAC is withheld or discontinued [37]. Despite this, contemporary evidence and expert consensus support that the net clinical benefit of anticoagulation favoring stroke prevention extends across the spectrum of frailty, and frailty should not be considered a contraindication to OAC therapy [38]. Frailty status is also dynamic and changes over months to years; serial reassessment in routine care may therefore add information that a single baseline measurement cannot.

Because both AF phenotype and anticoagulant prescribing can differ between men and women, we examined whether our associations were sex-dependent. In sex-stratified models the adjusted frailty–AF association reached significance in men but not in women, and the adverse-outcome signal appeared to express as bleeding in men and as hospitalization in women. However, formal interaction testing did not support a true sex difference for either the frailty–AF association (*p* = 0.33) or the AF–bleeding association (*p* = 0.30). The apparent divergence between sexes is therefore most consistent with reduced statistical power within sex-specific subgroups and the strong, sex-related distribution of anticoagulant use, rather than a genuine biological interaction. We accordingly interpret frailty as a sex-independent marker of risk for the course of AF, and present the sex-stratified estimates as exploratory (Supplementary Tables S1 and S2).

Pending confirmation in additional cohorts of older adults with AF, our findings raise the possibility that recurrent or persistent AF on follow-up ECG identifies patients at elevated bleeding risk through pathways only partially captured by current risk scores. The burden of AF is increasing, especially among older adults, and it is critically important that we refine our understanding of the epidemiology in patients treated with contemporary therapies. Our findings suggest that older patients with AF who are frail or pre-frail may require closer follow-up than non-frail individuals, and that nutritional or activity interventions to enhance physical functional status might mitigate risk of recurrent or persistent AF. Although frailty is associated with poorer clinical outcomes [39], frailty assessment is not typically part of routine clinical practice [40], in part because the standard frailty examination can require 20–40 minutes [41, 42]. Further studies are needed to evaluate whether components of the formal frailty assessment, such as grip strength testing [43], or automated electronic frailty scores derived from patient health records [44] might enable frailty assessment at scale across multiple clinic environments.

### Strengths & limitations

This study has several notable strengths. It uses detailed and highly curated clinical data from a relatively contemporary cohort of older adults; clinical outcomes and ECG data were rigorously adjudicated; and the two-year follow-up period was sufficient to examine recurrence or persistence of AF. Several limitations should be acknowledged. First, our operational definition of recurrence or persistence of AF progression based on transitions between paroxysmal, persistent, and permanent forms. We selected this operationalization deliberately because it reflects a common real-world clinical scenario in which a clinician interprets a single ECG at a routine clinic visit and again at follow-up; longitudinal clinical AF-type classification was not ascertained between visits in SAGE-AF, and a sensitivity analysis using the standard progression definition was therefore not feasible. Second, the snapshot ECG approach almost certainly misclassifies some participants with paroxysmal AF who are in SR at the moment of recording, a misclassification that would bias estimates toward the null. Third, outcomes were ascertained across the full follow-up period, but AF status was defined only at the year-two visit, so participants had to remain event-free until that visit to be classified—introducing immortal time and survivor bias that we could not address by modeling AF status as a time-dependent covariate, since longitudinal AF burden was not ascertained between visits. We accordingly interpret our outcome findings as consistent with, rather than as establishing, a contribution of recurrent or persistent AF to bleeding risk. Fourth, baseline AF type was missing for 12% of participants, which reduced the sample available for fully adjusted models; although AF type is an important confounder and was retained, this missingness is a source of potential selection bias. Fifth, residual confounding from health behaviors such as alcohol use, fall frequency, and repeat cardiovascular procedures cannot be excluded. Sixth, the sample primarily included white participants, which may limit generalizability. Finally, the two-year follow-up period may not have been sufficient to fully capture the impact of recurrent or persistent AF on relevant clinical outcomes.

## Conclusion

In this prospective study of 773 older adults with AF, baseline frailty or pre-frailty was significantly associated with higher odds of AF recurrence or persistence on ECG at a two-year follow-up, even after adjustment for traditional CV risk factors and treatments, with no significant difference by sex. Participants with recurrence or persistence of AF were at higher risk of major bleeding over the same period, although this association was sensitive to how anticoagulation was modeled. Our findings suggest that frailty may capture elements of risk for AF course over time in older adults that are not reflected in conventional risk scores. Further study is needed to determine whether assessment of frailty—including with scalable instruments—can enhance the management of older adults with, or at risk for, AF.

## Data Availability

The data used in this study are derived from the Systematic Assessment of Geriatric Elements in Atrial Fibrillation (SAGE-AF) cohort. Due to the inclusion of sensitive clinical and participant-level health information, the datasets are not publicly available in accordance with data use agreements and ethical regulations.

## Abbreviations

AF: Atrial fibrillation
AFEQT: Atrial Fibrillation Effect on Quality-of-Life questionnaire
BMI: Body mass index
CHA₂DS₂-VASc: Congestive heart failure, hypertension, age ≥ 75 years (doubled), diabetes mellitus, stroke or transient ischemic attack (doubled), vascular disease, age 65–74 years, sex category (female)
CHS: Cardiovascular Health Study
CKD: Chronic kidney disease
CRNM: Clinically relevant non-major
DOAC: Direct-acting oral anticoagulant
ECG: Electrocardiogram
GAD-7: Generalized Anxiety Disorder-7
GEE: Generalized estimating equations
HF: Heart failure
IRB: Institutional Review Board
MI: Myocardial infarction
MoCA: Montreal Cognitive Assessment
NVAF: Non-valvular atrial fibrillation
OAC: Oral anticoagulant
PHQ-9: Patient Health Questionnaire-9
PVD: Peripheral vascular disease
SAGE-AF: Systematic Assessment of Geriatric Elements in Atrial Fibrillation
SR: Sinus rhythm
VKA: Vitamin K antagonist
